# Efficacy of a patient with scar constitution combined with corrosive esophageal strictures after different endoscopic therapies

**DOI:** 10.1002/ccr3.8156

**Published:** 2023-11-17

**Authors:** Haixia Wang, Wei Tao

**Affiliations:** ^1^ Department of Gastroenterology Zigong First People's Hospital Zigong China; ^2^ Department of Gastroenterology General Hospital of Ningxia Medicale' Yinchuan China

**Keywords:** corrosive esophageal stricture, dilatation, endoscopic radial incision, endoscopic therapy, stent placement

## Abstract

Oral corrosive substances can cause esophageal or gastrointestinal strictures, leading to malnutrition and gastrointestinal dysfunction, directly affecting the patients' quality of life. The aim of the case was to compare the efficacy of different endoscopic therapy methods.

## Graphical abstract

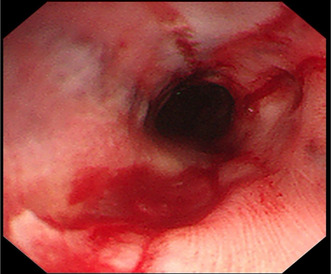

## 1 INTRODUCTION

Corrosive esophagitis is characterized by caustic injury due to the ingestion of chemical agents, mainly alkaline substances such as detergents, cleaning compounds, and bleach.^1^ The severity of the injuries caused by caustic ingestion depends on gastric contents and caustic agent properties, including pH, concentration, ingested volume, and tissue contact duration.^2^ In general, tissues are more severely damaged by alkaline solutions than by acidic solutions. This is because alkalis cause liquefactive necrosis, while acids cause coagulative necrosis resulting in eschar formation, thereby protecting the mucosal epithelium from deep ulcer formation. High‐degree corrosive esophagitis may result in esophageal bleeding, perforation, and even death in the worst cases. In particular, esophageal strictures are one of the more serious complications of corrosive esophagitis. Corrosive strictures are frequently very tight, long, eccentric, and multiple, and may require recurrent endoscopic therapy procedures, which are costly. There is a belief that these patients have a very high stricture recurrence rate because of extensive esophageal damage and fibrosis. For these reasons, patients with corrosive strictures often undergo surgery in the past, which carries significant morbidity and mortality. But now, early endoscopic therapy is very useful in cases of corrosive esophageal strictures (CES), not only for determining the degree of mucosal injury and severity but also for predicting prognosis.^3^

Current endoscopic management for CES includes esophageal dilatation, stent placement, endoscopic incision, injection of steroids into the stricture segment, and so on.^4^ The foremost goal of therapy for CES is to preserve the esophagus and restore its function. Dilatation has been considered as the therapy of choice for CES and can be performed endoscopically, using a balloon dilator or rigid dilator. Esophageal stent placement or endoscopic incision is indicated when dilatation is not successful. Several case series reports have shown that endoscopic therapy is a safe and effective therapy for CES.^5^, ^6^, ^7^ However, there is still a lack of a well‐established consensus on when and how to optimize endoscopic therapy for CES. Here, we report the case of a male Chinese patient with CES with scar constitution due to the ingestion of caustic soda that achieved a favorable outcome after repeated different endoscopic therapies to compare the safe and effectiveness of different endoscopic therapies.

## 2 CASE

**FIGURE 1:**
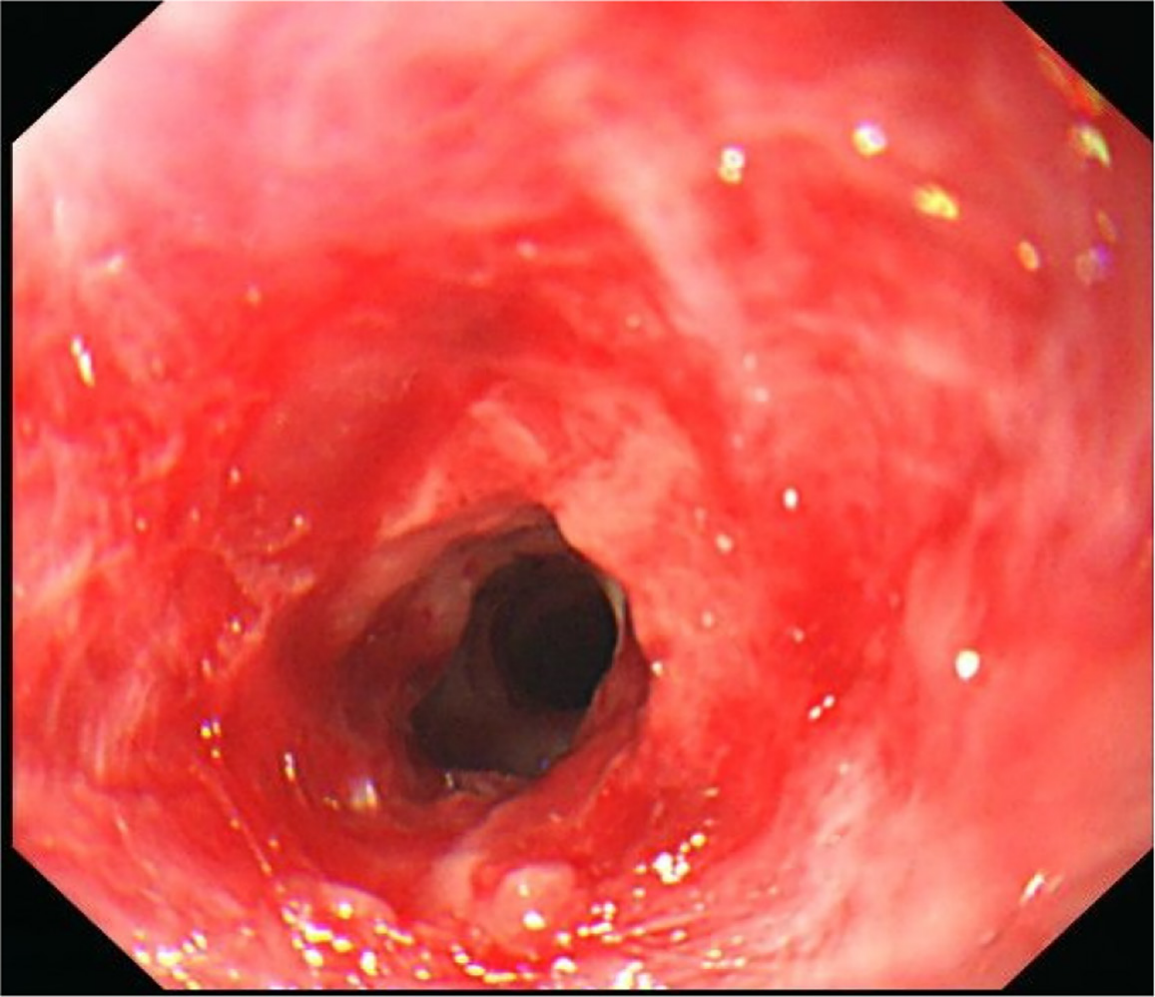
The patient underwent upper gastrointestinal endoscopy on Week 4 after admission, and it was concordant with the grade IIb.

A 36‐year‐old male ingested approximately 100 mL of caustic soda liquid (sodium hydroxide) in a suicide attempt. He presented to our emergency department after 2 h on November 13, 2016, with nausea, vomiting, retrosternal pain, hematemesis (approximately 100 mL), and dyspnea. He was belonging to the scar constitution. Upon admission, physical examination was positive for tachycardia, tachypnea, and coarse respiratory sounds. Body temperature was 37.9°C, and blood pressure was 108/62 mmHg. Oropharyngeal injury and hemorrhagic, however, his abdomen was flat and soft. Bowel sounds were normal. Although marked epigastric tenderness was present, there was no rebound tenderness or guarding. His complete blood count and biochemistry tests were in normal range, except for a white blood cell count of 20.93 × 10^9^/L, neutrophils 16.94 × 10^9^/L, and neutrophil percentage 80.9%. Contrast‐enhanced computed tomography (CT) chest and CT of the whole abdomen revealed no esophageal or gastrointestinal perforations. During hospitalization, the major short‐term complications of pneumonia, hemorrhage, and airway obstruction for the patient. His therapy included tracheotomy, intravenous fluid, total parenteral nutrition, H2 receptor blockers, antibiotherapy, and other symptomatic therapies. In addition, he can drink water without choking and coughing until Day 10, and was allowed to eat fluids until Week 2. Initial endoscopy was withheld because there was a high risk of esophageal or gastric perforation. Besides, the patient underwent upper gastrointestinal endoscopy on Week 4 after admission revealed multiple serious strictures in the whole of the esophagus (Figure 1). It was concordant with the grade IIb of the caustic esophageal injury classification defined by Zargar et al.^8^ Finally, the patient was diagnosed with CES caused by sodium hydroxide. He is a scar constitution, surgery was traumatic and risky, and the patient refused to undergo surgical therapy, so he was fed by nasogastric tube.

**FIGURE 2:**
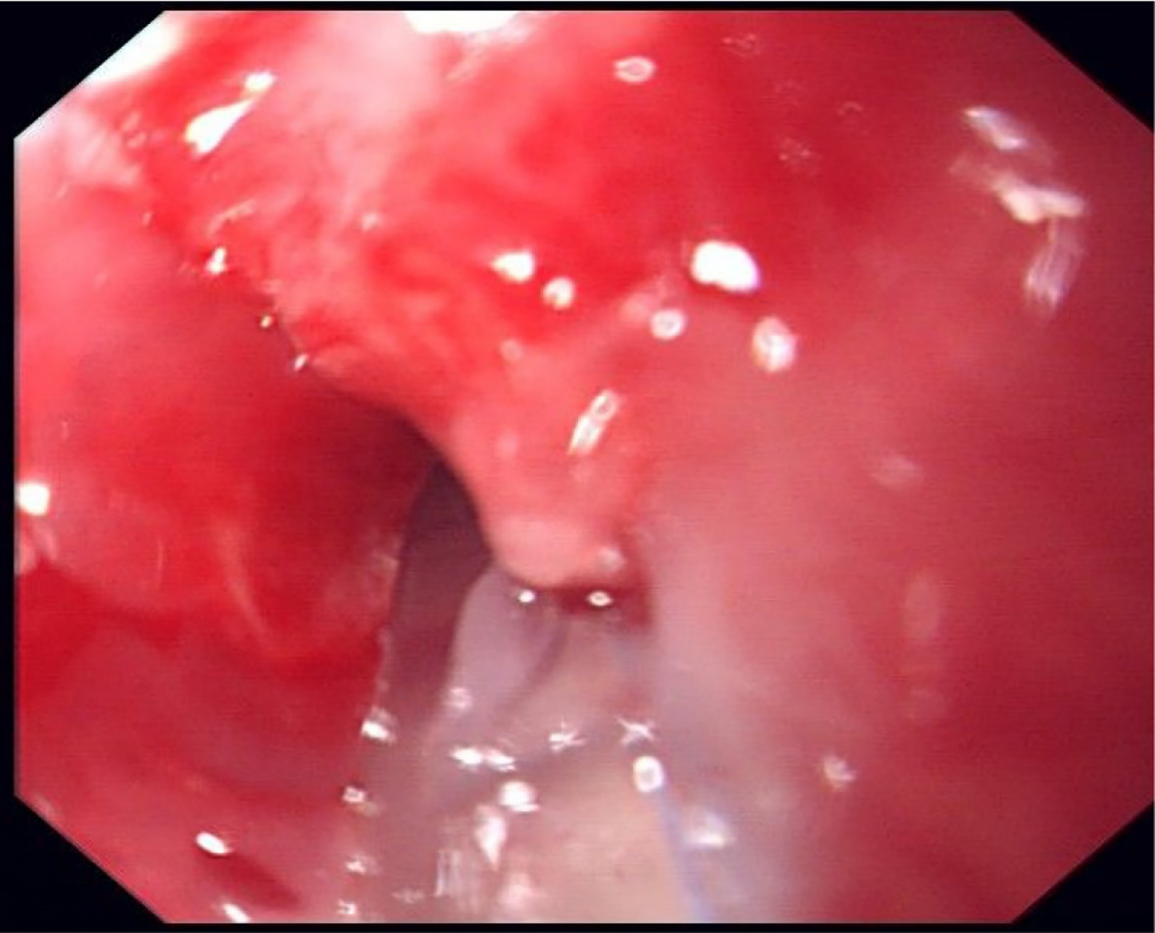
The patient underwent a gastroscopy on 11 weeks.

**FIGURE 3:**
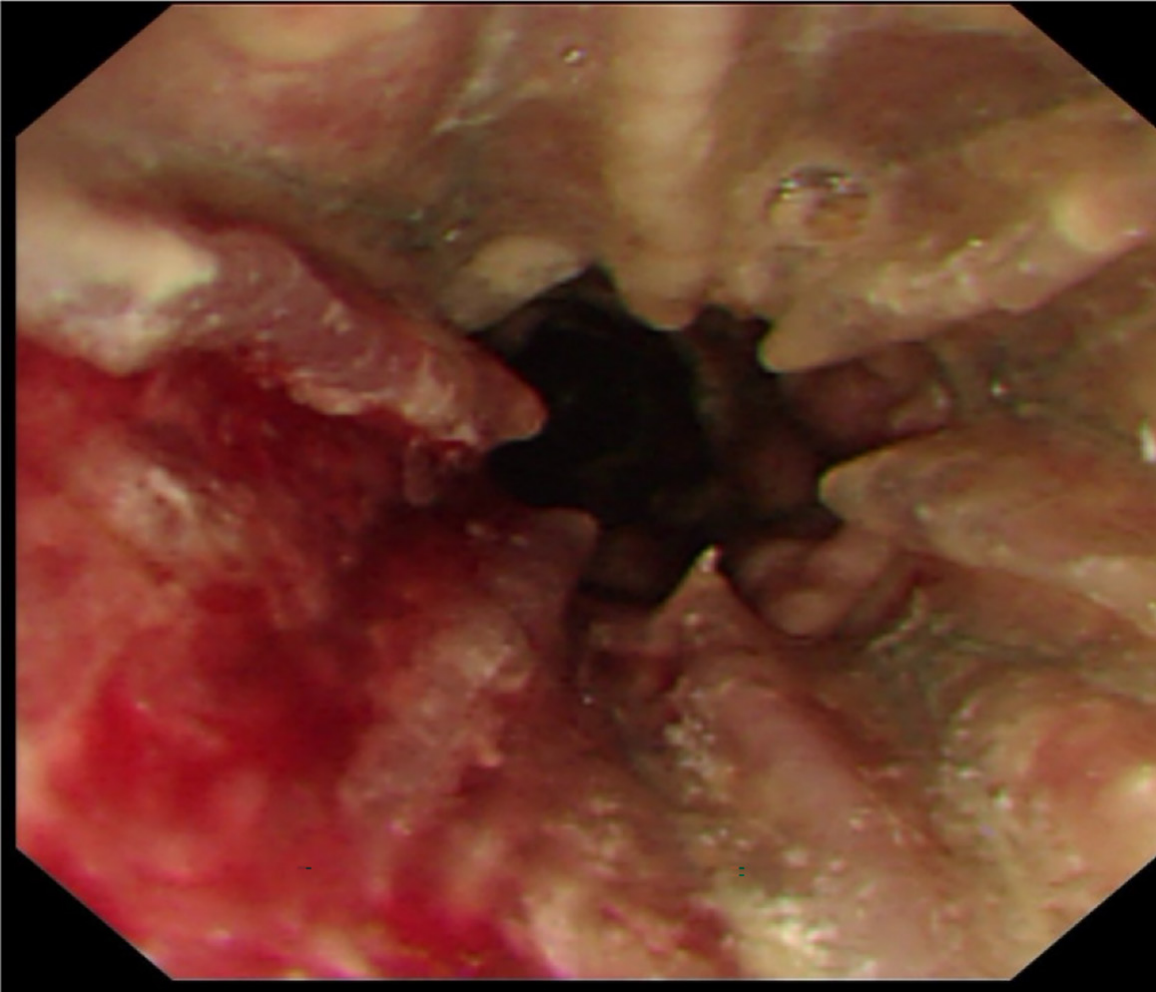
The implanted stent could not take out due to the severe scar hyperplasia.

**FIGURE 4:**
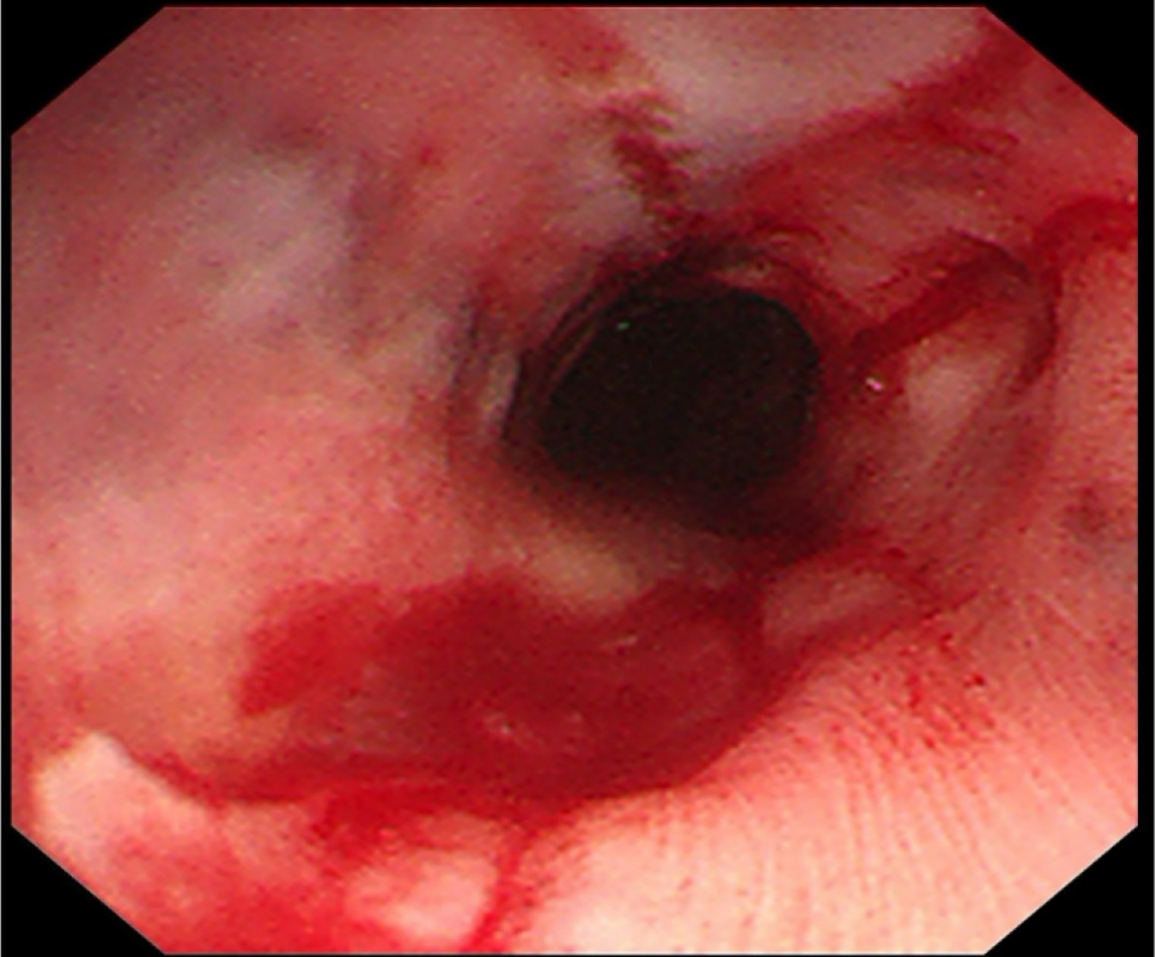
The patient achieved a favorable outcome finally.

Eleven weeks later, the patient underwent a gastroscopy (Figure 2), which found that “a stenotic segment was visible at a distance of 16–20 cm from the hilar tooth and the gastroscope (Olympus GIFXP260: 9 mm) could not be passed”. After absolute contraindications were ruled out, the patient was treated with careful serial graded dilatations by selecting an initial dilator size based on the estimated stricture diameter (diameters of 7, 9, 11, 13, and 15 mm), the number of bougies ≤3 at a time. From January 22, 2017, to February 19, 2017, the patient used to be handled with weekly dilatation per week, with unfavorable outcomes after five therapies. Esophageal stent placement or ERI was indicated when dilatation is not successful. After that, the patient was treated endoscopically if the Stooler^9^ grade of his dysphagia was ≥2. From February 26, 2017 to April 19, 2018, The patient underwent 4 times stent placement (the average remission time of his dysphagia was 47.25 days) combined with intermittently dilatation 13 times (the average remission time was 18.85 days) to improve esophageal dysphagia. Due to scar constitution, stent migration, stent dislocation, and so on, the patient still had recurrent dysphagia. So from May 17 to September 27, 2018, the patient underwent endoscopic radial incision (ERI) for 4 times (the average time of remission was 59.75 days) while taking oral steroid (the initial amount was 30 mg Qd, and the amount was reduced by 5 mg every 2 weeks until the drug was stopped). During the period of oral steroid, the patient was treated with ERI 3 times and once stenting, the mean remission time was 33.25 days and once ERI after therapy his dysphagia improved significantly. However, the patient reoccurred dysphagia grade 3; again after 5 months, he chose dilatation 6 times again from February 24, 2019, to September 15, 2019 (the average remission time was 37.83 days), and then, he was stented 1 time (the remission time was 98 days). Implanted stent were difficult to take out several times due to the severe scar hyperplasia (Figure 3), so that the patient was continued to be maintained on dilatation 6 times (the average remission time was 127.33 days). The patient had no similarly esophageal strictures and able to eat solid food at 2 years of follow‐up. The therapy has achieved a favorable outcome (Figure 4).

The patient underwent endoscopic dilatation therapy 30 times, stent placement 6 times, ERI 4 times, and oral steroid for 3 months during the 6‐year course of the disease. The number of therapies per year decreased with the duration of the disease, and the remission time to dilatation gradually increased. ERI had the longest average remission time among the four therapy modalities and the shortest remission time for oral steroid (Table 1).

**TABLE 1:** Remission time after different endoscopic therapies.

	The number of therapies [*n*]	*x* ± *s* (day)
Dilatation	30	45.84 ± 61.27
Stent placement	6	52.33 ± 30.32
ERI	4	59.75 ± 59.61
Endoscopic therapies during oral steroid	4	33.25 ± 7.41

A favorable outcome was defined as patients being able to swallow solid or semisolid food and maintain a nutritional status for ≥6 months without any endoscopic therapy or surgical intervention.^2^

## 3 DISCUSSION

Corrosive esophagitis is characterized by caustic injury following the ingestion of chemical agents. Esophageal bleeding, perforation, or strictures can be worsened by high‐degree corrosive esophagitis. Alkalis substances may cause severe post‐corrosive injury of the upper gastrointestinal tract, including perforation that often results in death. The most common complications are esophageal and gastric strictures, which are found in greater percentages than in poisonings with acid substances. Strictures of the esophagus may appear 3 weeks after ingestion of the corrosive substance, in the first 3 months, or according to some authors, even after 1 year following caustic ingestion.^10^ Liquid corrosive substance ingestion more often initiates stenosis than corrosive substances in crystal form. Currently, the majority of endoscopists recommend early endoscopy to determine the severity and extent of the injury and thereby predict prognosis. However, comprehensive guidelines informing the timing and patient selection for endoscopy following ingestion of caustic agents have yet to be established. The timing recommended in the literature remains particularly controversial. Numerous studies recommend that the most optimal timing for esophagogastroduodenoscopy is the first 12–24 h post‐ingestion.^11^ Conversely, other studies recommend endoscopy should be performed between 24 and 48 h as the damage will have yet to mature before 12 h and will therefore be underestimated by examination. Since the initiation of fibroplasia, inflammatory changes, and the healing process of the post‐corrosive injuries begin on the fifth and are most intensive until the 15th day, it is suggested to avoid endoscopy during this period.

Scarring stricture is very difficult to treat in clinical practice. Esophageal or gastric strictures can cause many symptoms such as dysphagia and malnutrition, which seriously affects the quality of patients' life.^12^, ^13^ It is especially important to choose the appropriate therapy plan in time to reduce complications and mortality. In recent years, endoscopic therapy has become the optimal therapy method for benign esophageal strictures. Corrosive esophageal stricture is common with complex stenosis. For esophageal strictures, endoscopic therapies include dilatation, stent placement, endoscopic dissection, medication, and cell transplantation, all of which can relieve dysphagia.

Benign esophageal stricture using different endoscopic therapy is considered safe and effective for both short‐ and long‐term relief of dysphagia. Endoscopic therapy, rather than surgery, has therefore been suggested as the primary therapy for most of these patients. The reported experience is mostly in patients with peptic strictures, and there is little information on the efficacy and safety of different endoscopic therapies in patients with CES. Due to this lack of formal criteria, we are still unable to clarify how to choose the optimal endoscopic therapy method based on the situation of patients with benign esophageal strictures. Many scholars suggested that the decision to select the type of endoscopic therapy is based on the assessment of stricture (simple vs. complex), the length and distal extent of the stricture, and the experience of the endoscopist.^14^ Here, we describe the first case of a patient who ingested sodium hydroxide and consequently presented with CES; moreover, he was treated with multiple endoscopic methods. Since the esophageal strictures were located in the upper and high risk of surgery, the patient chose endoscopic therapy. During the 6‐year period, by comparing the remission time of these four endoscopic therapies, we found that ERI had the longest remission time, and the shortest was oral steroid, and the remission time continued to lengthen as the number of therapies increased, besides the therapeutic efficacy became increasingly favorable.

In this case, the patient was initially treated with bougie dilatation. During the dilatation process, we started with a 7‐mm bougie according to the degree of stenosis, and then increased the diameter of dilatation step by step to expand the stenosis to the maximum extent possible, and then decided to terminate the diameter of the bougie according to the patient's condition. We found that the strict implementation of these procedures helped prevent complications and avoid restenosis after therapy. The shortest remission time with oral steroid therapy may be related to the Koebner^15^ phenomenon, which can induce ulcers, affect wound healing, and let it to proliferate faster since scar constitution. For complex benign esophageal strictures, ERI has been observed to have a longer remission time and better efficacy than other endoscopic therapies in clinical practice and can be the therapy of choice.

Complex esophageal stricture usually require numerous endoscopic therapies. There is no consensus endpoint for endoscopic therapy, and which is the optimal therapy.^4^ Some person suggested that endoscopic dilatation is the first choice for certain peptic strictures. But for complex strictures, dilatation cannot offer durable remission, so the requirement for multiple dilatation treatment sessions. Stenting or endoscopic incision may be performed in patients with complex stenosis who achieve an unfavorable outcome with repeated dilatation. A reported that stents achieved a satisfactory improvement/resolution of the refractory strictures with a success rate of 35%–45%; however, migration rates (25%–35%) and adverse events (20%–25%) are fairly common.^16^ A study by Manabu Muto et al.^17^ showed that radial incision and cutting is an effective and safe method for gastroesophageal anastomotic strictures that are refractory to repeated endoscopic dilation, at the same time radial incision and cutting also avoided perforation and bleeding, which is consistent with our conclusion. For CES, the use of steroids remains controversial. Using steroids is believed to inhibit the inflammatory response and consequently reduce the stricture formation. Studies have shown that the use of steroid injections, in conjunction with antisecretory therapy and dilations, reduces the number of repeat dilations and increases the dilation‐free period,^18^, ^19^ but the effectiveness of using steroids was not satisfactory in the case. Therefore, we believe that the preferred choice of steroids for CES is not recommended.

In summary, complex esophageal stricture due to corrosive esophagitis combined with scar constitution is difficult to treat, and a favorable outcome can be achieved after numerous endoscopic therapies. What's more, ERI and occasionally stents can provide durable remission for such patients, which is safe and effective in clinical practice. But more better modalities can provide durable efficacy need to be investigated further.

## AUTHOR CONTRIBUTIONS

**Haixia Wang:** Data curation; formal analysis; software; visualization; writing – original draft; writing – review and editing. **Wei Tao:** Conceptualization; methodology; resources; supervision.

## FUNDING INFORMATION

No funding.

## CONFLICT OF INTEREST STATEMENT

The authors declare that they have no conflict of interest.

## ETHICS STATEMENT

The principles outlined in the Declaration of Helsinki was followed. The subject of the case report provided informed consent to publish the included information.

## CONSENT

Written informed consent was obtained from the patient to publish this report in accordance with the journal's patient consent policy.

## PATIENT ANONYMITY AND INFORMED CONSENT

Obtained informed consent from the patient.

## Data Availability

All relevant data are within the paper.
